# A Report of a Rare Case of Choledocholithiasis Complicated by Proximal Basket Rod Rupture Requiring Surgical Intervention

**DOI:** 10.7759/cureus.82357

**Published:** 2025-04-16

**Authors:** Alexandre Gomes

**Affiliations:** 1 Department of Surgery, Faculdade de Ciências Médicas e da Saúde, Pontifícia Universidade Católica de São Paulo (FCMS-PUCSP), Sorocaba, BRA

**Keywords:** cholangiopancreatography, choledocholithiasis, laparotomy, lithotripsy, surgery

## Abstract

We report a rare case of choledocholithiasis in which mechanical lithotripsy led to rupture of the proximal rod of the Dormia basket, complicating the removal of calculi via endoscopic retrograde cholangiopancreatography (ERCP) and necessitating surgical intervention. ERCP plays a key role in the management of biliopancreatic disorders. Although this approach has improved in recent decades, it remains one of the endoscopic procedures with the highest complication rates. These complications may arise due to the presence of large or impacted stones, resulting in excessive stress on the basket wires or rod. Basket impaction may lead to several complications, such as cholangitis, common bile duct (CBD) perforation, and pancreatitis. Therefore, early removal of the impacted device is recommended. However, no ideal technique for its removal has been established. A 34-year-old female patient presented with pain in the upper right quadrant of the abdomen and jaundice, but no fever. Imaging findings revealed cholelithiasis and choledocholithiasis. ERCP showed 10-mm calculi in the proximal segment of the CBD, along with a distal long-segment narrowing. Mechanical lithotripsy was unsuccessful in fragmenting the calculi, and a complication occurred when the proximal rod of the basket broke, making it difficult to remove the basket along with the trapped calculi. A biliary plastic stent was placed. The endoscopist suggested delayed removal of the basket, stone, and stent. Nevertheless, the attending surgeon opted for open surgery two days later, performing a cholecystectomy and removal of the stent, calculi, and basket via duodenotomy.

## Introduction

Impaction of a lithotripter basket with an entrapped stone or rupture of the traction wire during mechanical lithotripsy occurs in 0.8-6% of procedures [1]. Few cases with similar presentations have been described in the medical literature, with resolution achieved through laparoscopy or laparotomy, but we found no case like the one described here [2-5]. Currently, 85-90% of choledocholithiasis cases are successfully treated by endoscopic retrograde cholangiopancreatography (ERCP) techniques: papilla sphincterotomy and basket or balloon extraction. Mechanical lithotripsy captures the stone with a retrieval basket and applies force against a metal sheath to fracture it. The technique has reported success rates of up to 90%, with a low complication rate. The most common complications include basket or system fracture with stone retention, pancreatitis, and bleeding [6].

## Case presentation

**Figure 1:**
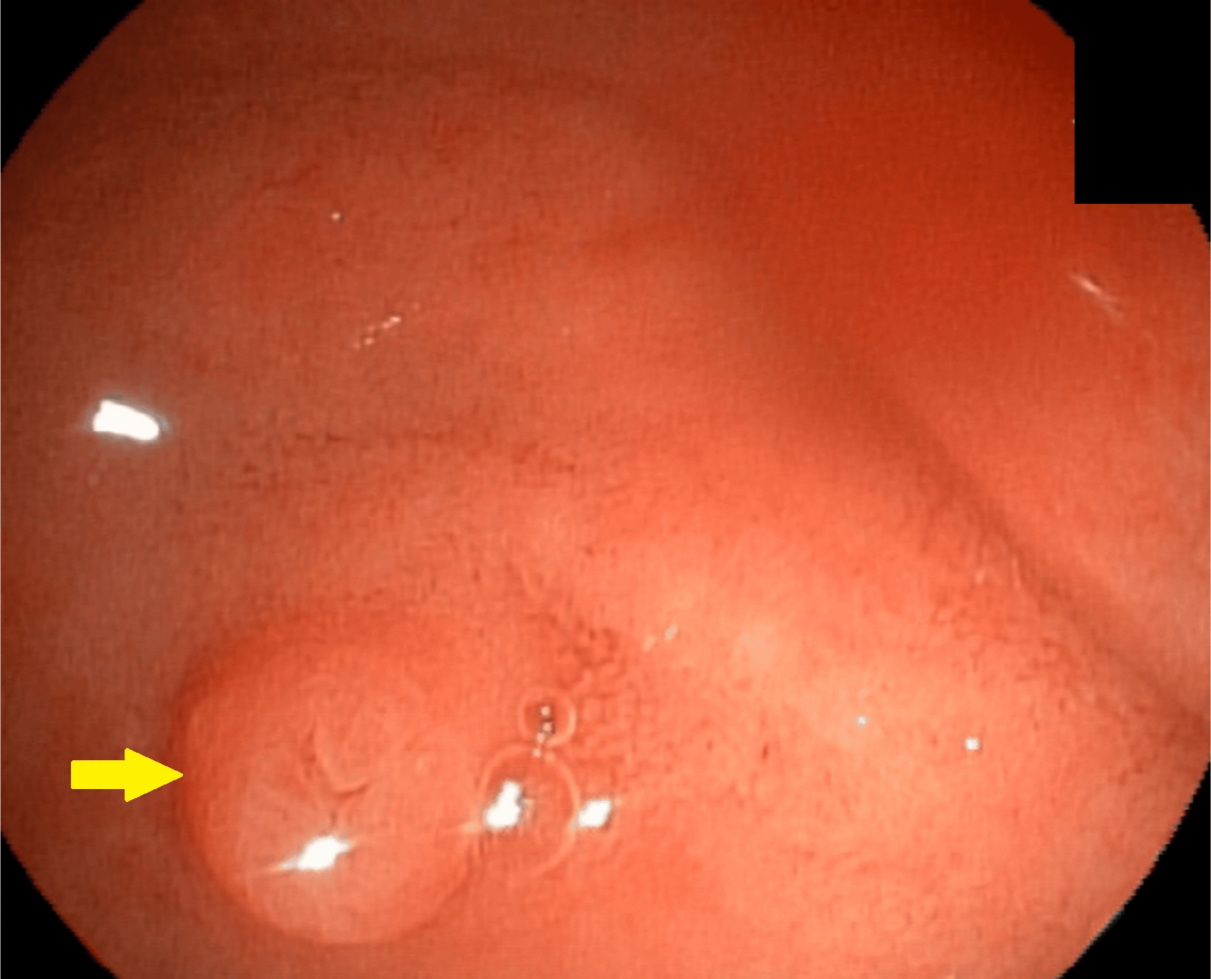
Small, flat-type papilla without protrusion, with difficult cannulation

**Figure 2:**
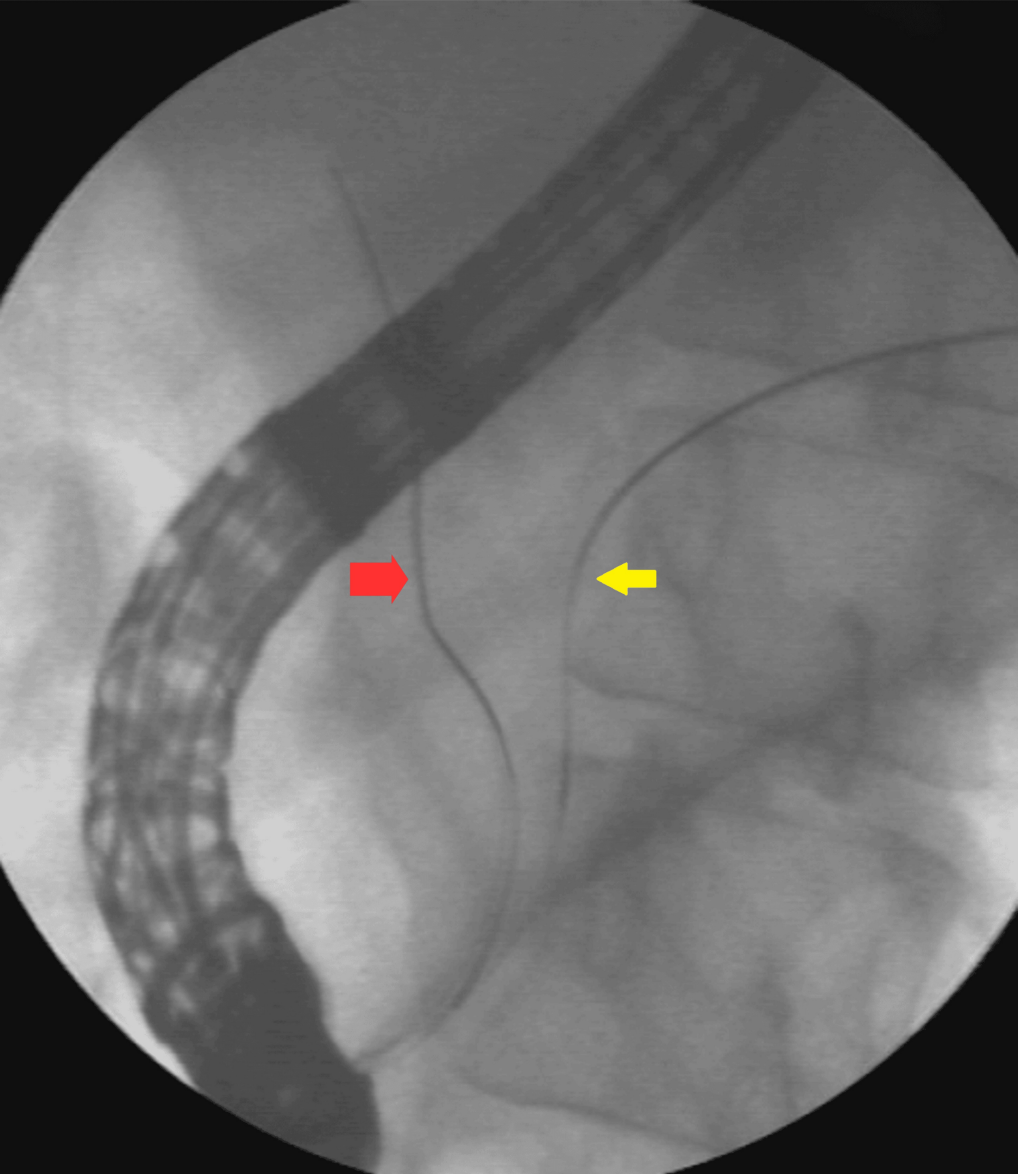
Double-wire cannulation technique The first guide wire (yellow arrow) went to the main pancreatic duct, and the second wire (red arrow) went to the CBD. CBD: common bile duct

A 34-year-old white female patient with a body mass index (BMI) of 35 presented with right upper quadrant abdominal pain and jaundice, without fever. Imaging studies revealed cholelithiasis and choledocholithiasis. During ERCP, a small papilla of Vater was identified, making cannulation of the common bile duct (CBD) difficult; the guidewire repeatedly entered only the main pancreatic duct. The double-wire cannulation technique was therefore employed, allowing successful access to the bile duct (Figures 1-2).

**Figure 3:**
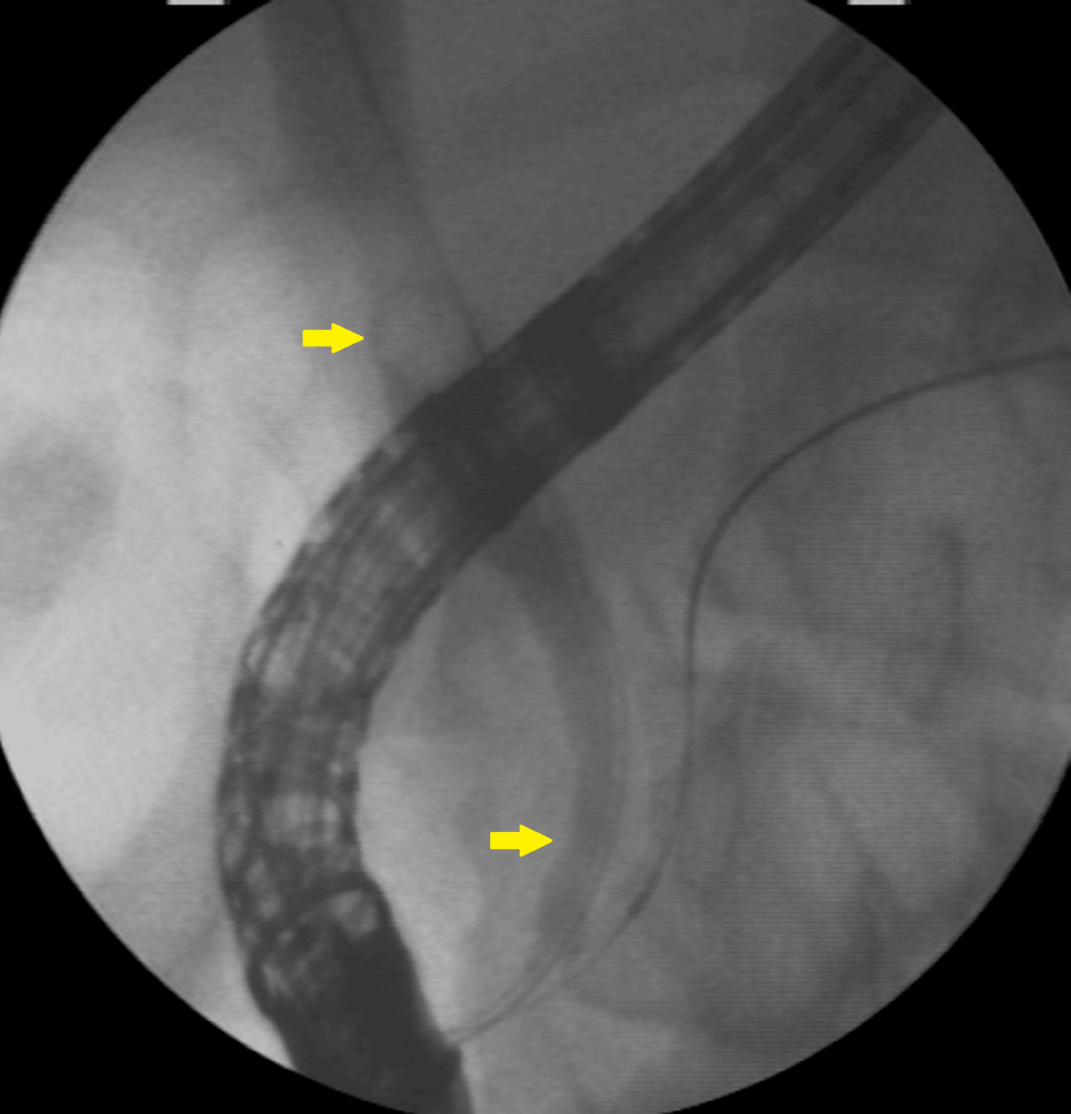
Radioscopy showing CBD with calculi and distal narrowing A 10-mm stone in the proximal CBD and long narrowing of the distal CBD. CBD: common bile duct

Imaging revealed 10-mm calculi in the dilated proximal CBD and a long narrowing of the distal CBD (Figure 3).

**Figure 4:**
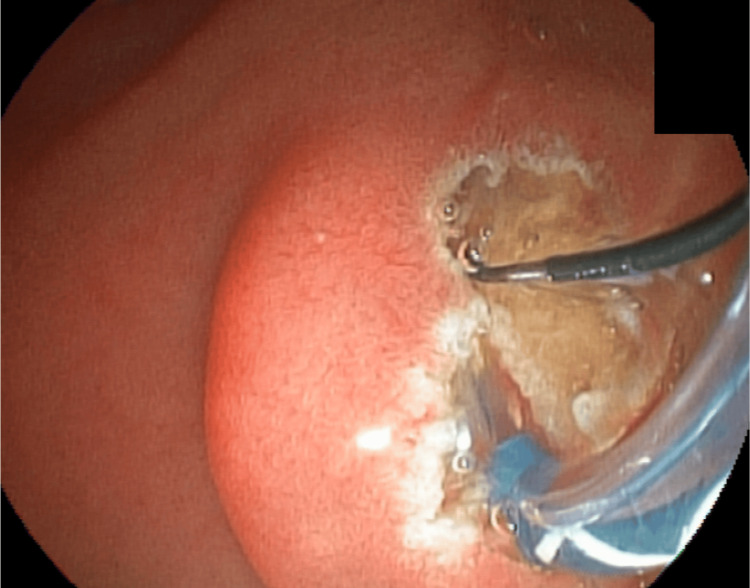
Sphincterotomy

**Figure 5:**
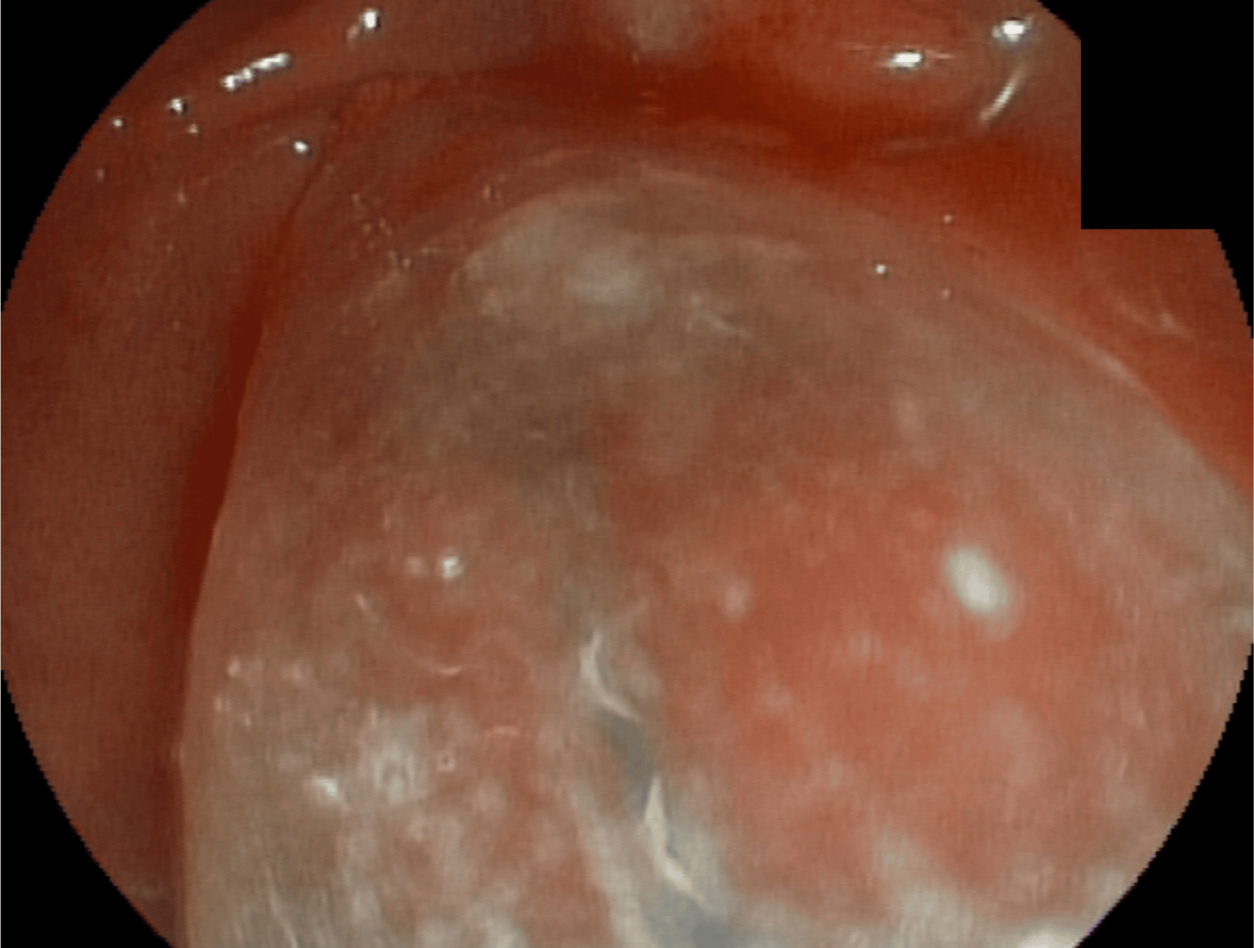
Dilation of the distal CBD with a 15-mm balloon catheter CBD: common bile duct

A sphincterotomy was performed (Figure 4), followed by dilation of the distal CBD using a 15-mm balloon catheter (Figure 5).

**Figure 6:**
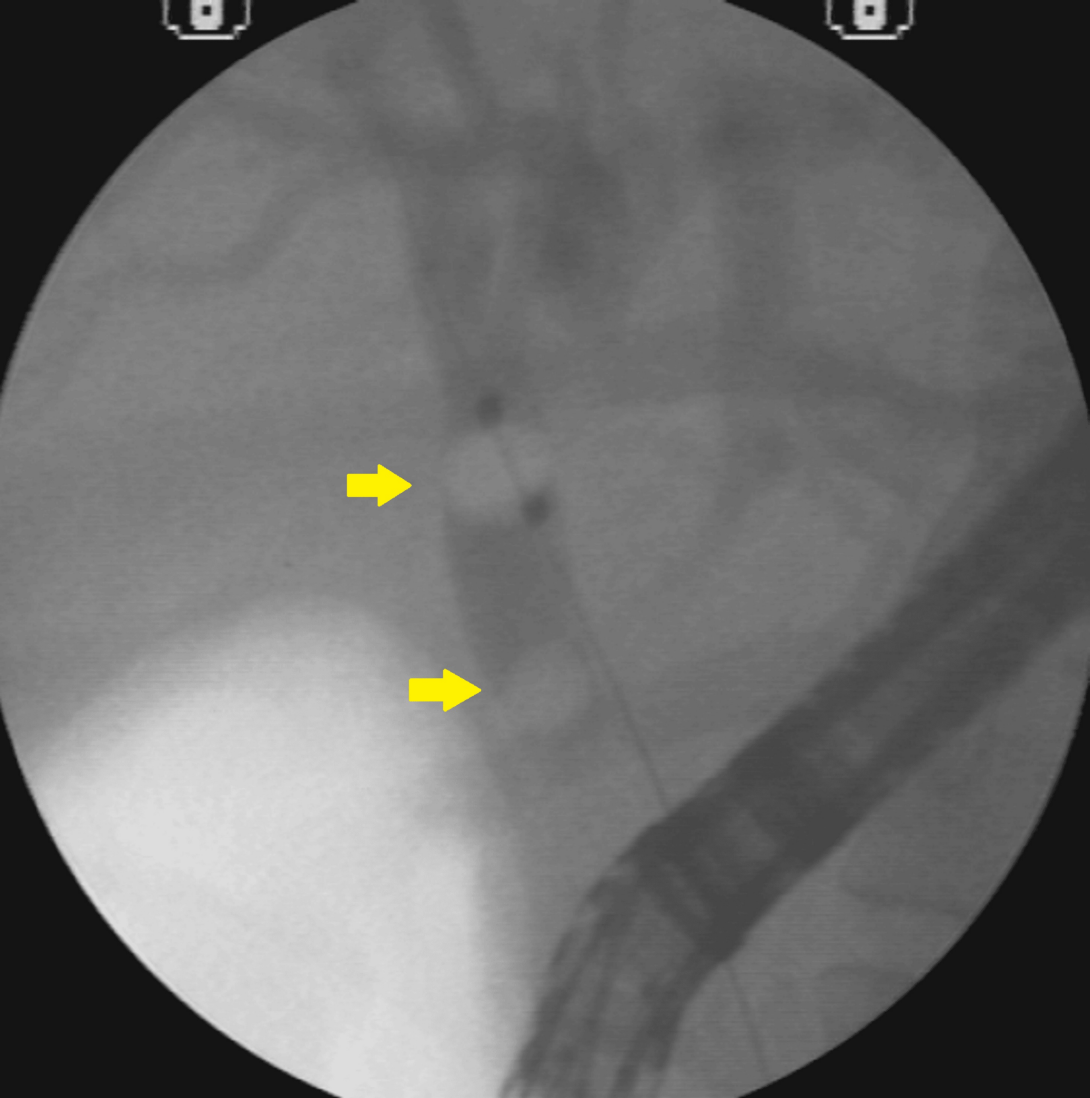
Calculi and stone extraction balloon The stone was not removed due to the distal CBD disproportion. CBD: common bile duct

However, stone removal with a stone-extraction balloon was unsuccessful (Figure 6).

**Figure 7:**
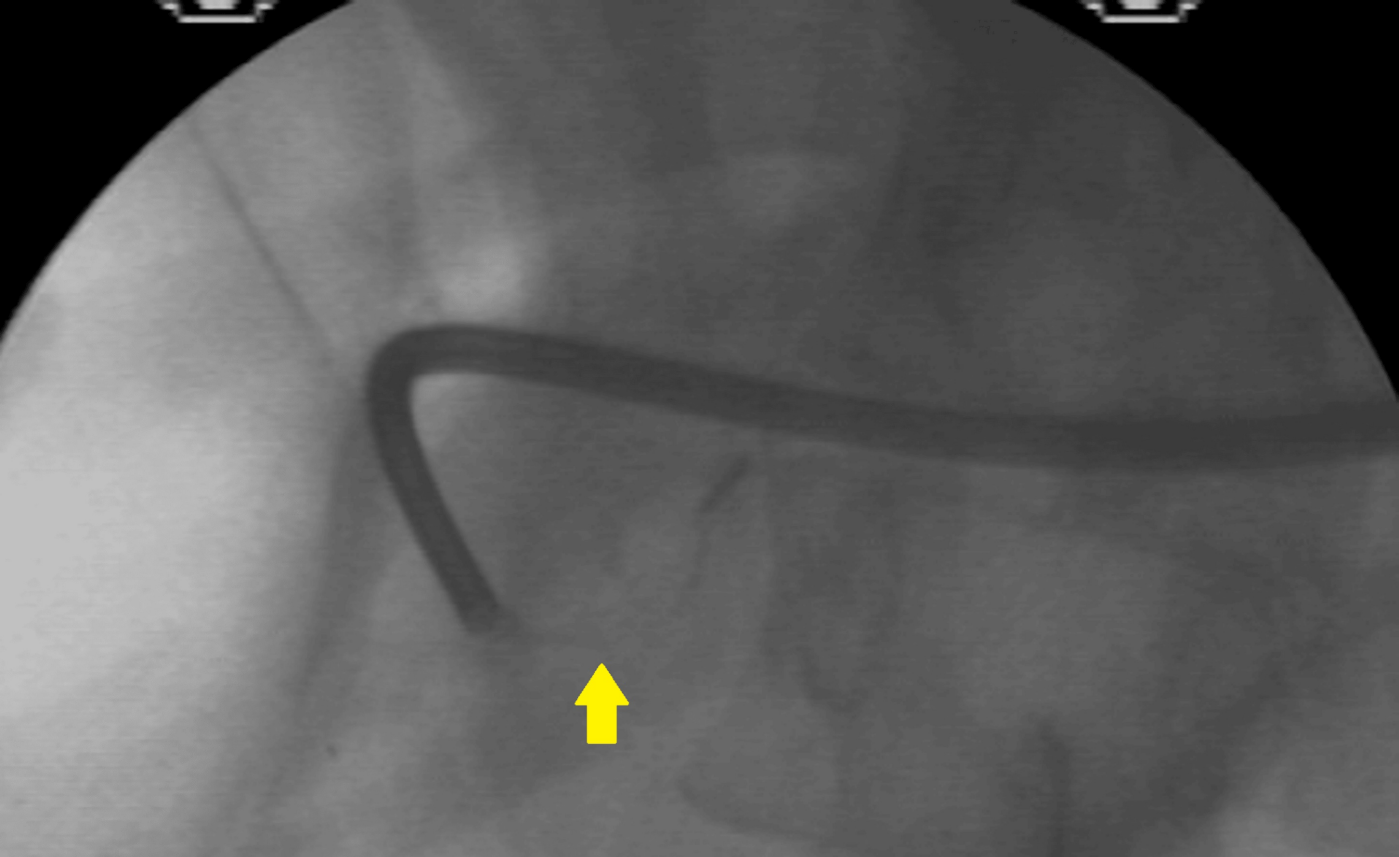
Mechanical lithotripsy

Basket passage and stone capture for attempted removal; however, distal CBD narrowing did not allow the set to be removed. Mechanical lithotripsy was performed in an attempt to fragment the stone (Figure 7).

**Figure 8:**
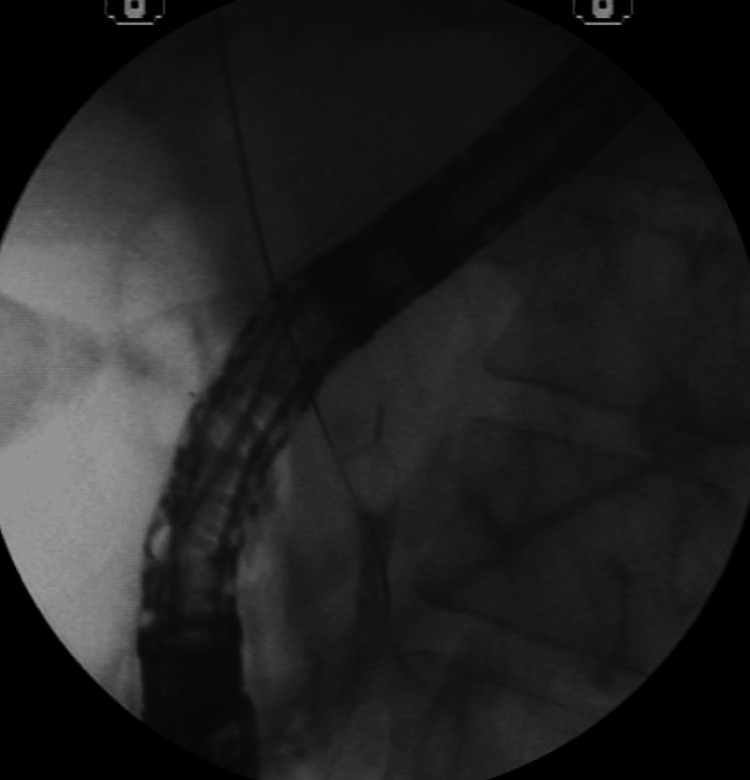
Dormia along the entrapped stone and the broken rod

Mechanical lithotripsy was unsuccessful in fragmenting the stone, and a complication arose when the proximal rod of the basket broke, making it challenging to remove the basket along with the trapped stone (Figure 8).

**Figure 9:**
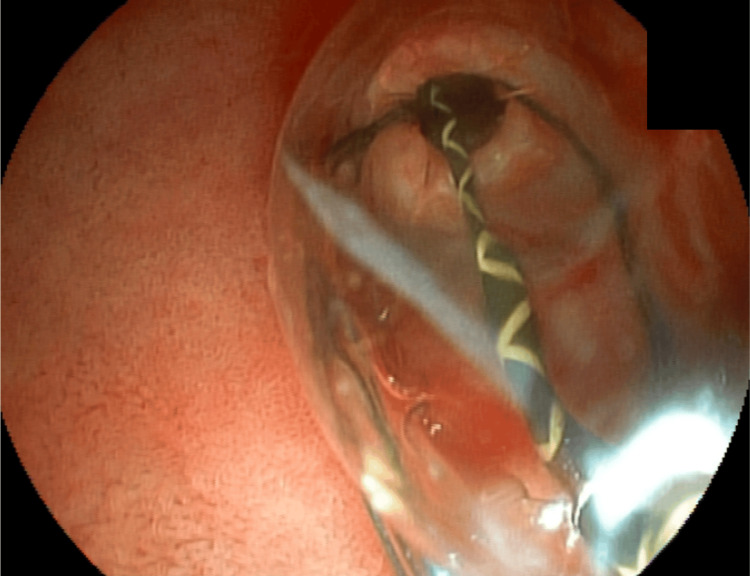
Second dilation of the distal CBD with a 15-mm balloon catheter CBD: common bile duct

A second 15-mm dilation was performed to remove the imprisoned basket along with the calculi (Figure 9).

**Figure 10:**
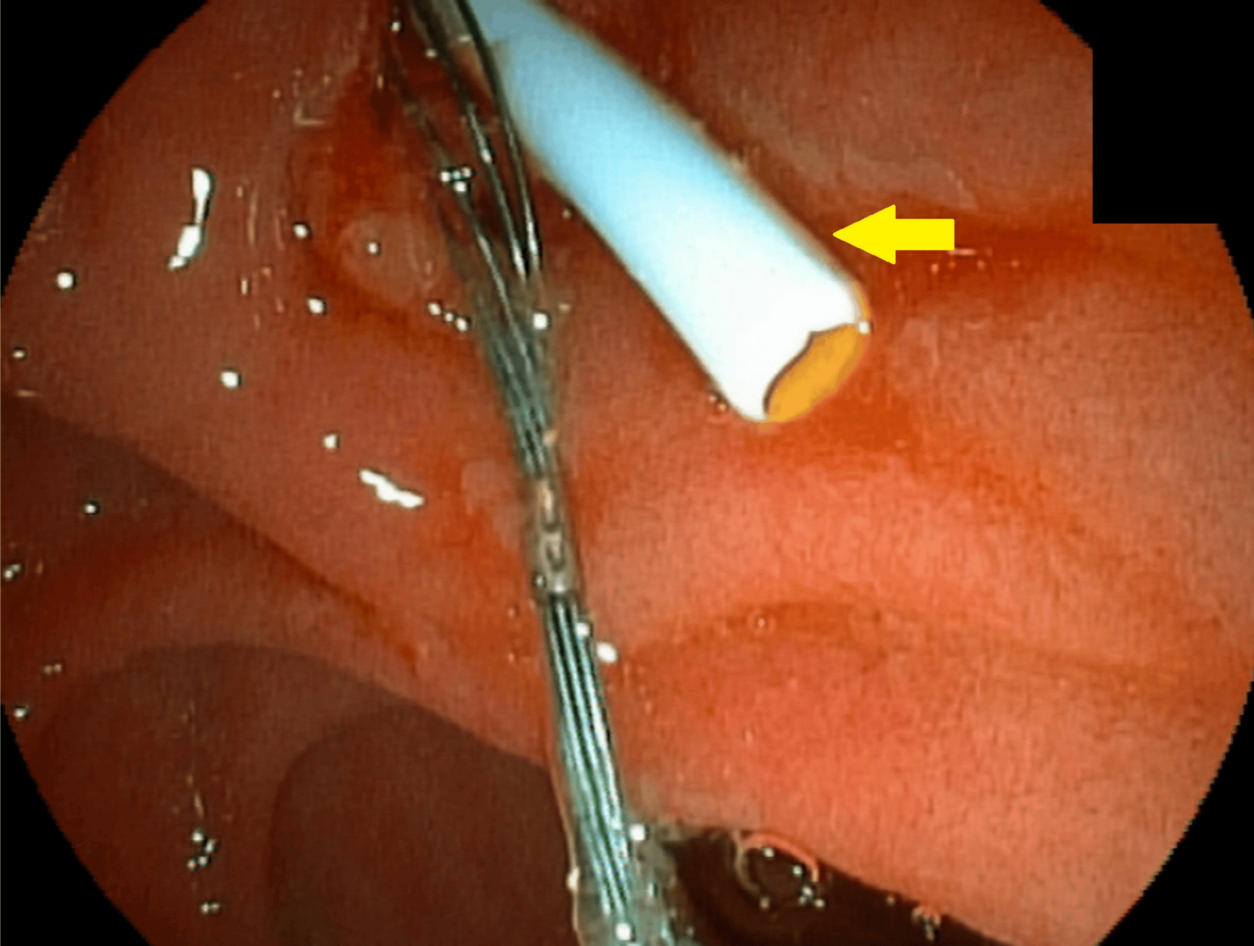
Placement of the 8.5-French biliary plastic stent

After this new dilation, the removal of the incarcerated set was attempted using mouse tooth forceps. However, no success was noted. An 8.5-French biliary plastic stent was placed (Figure 10).

**Figure 11:**
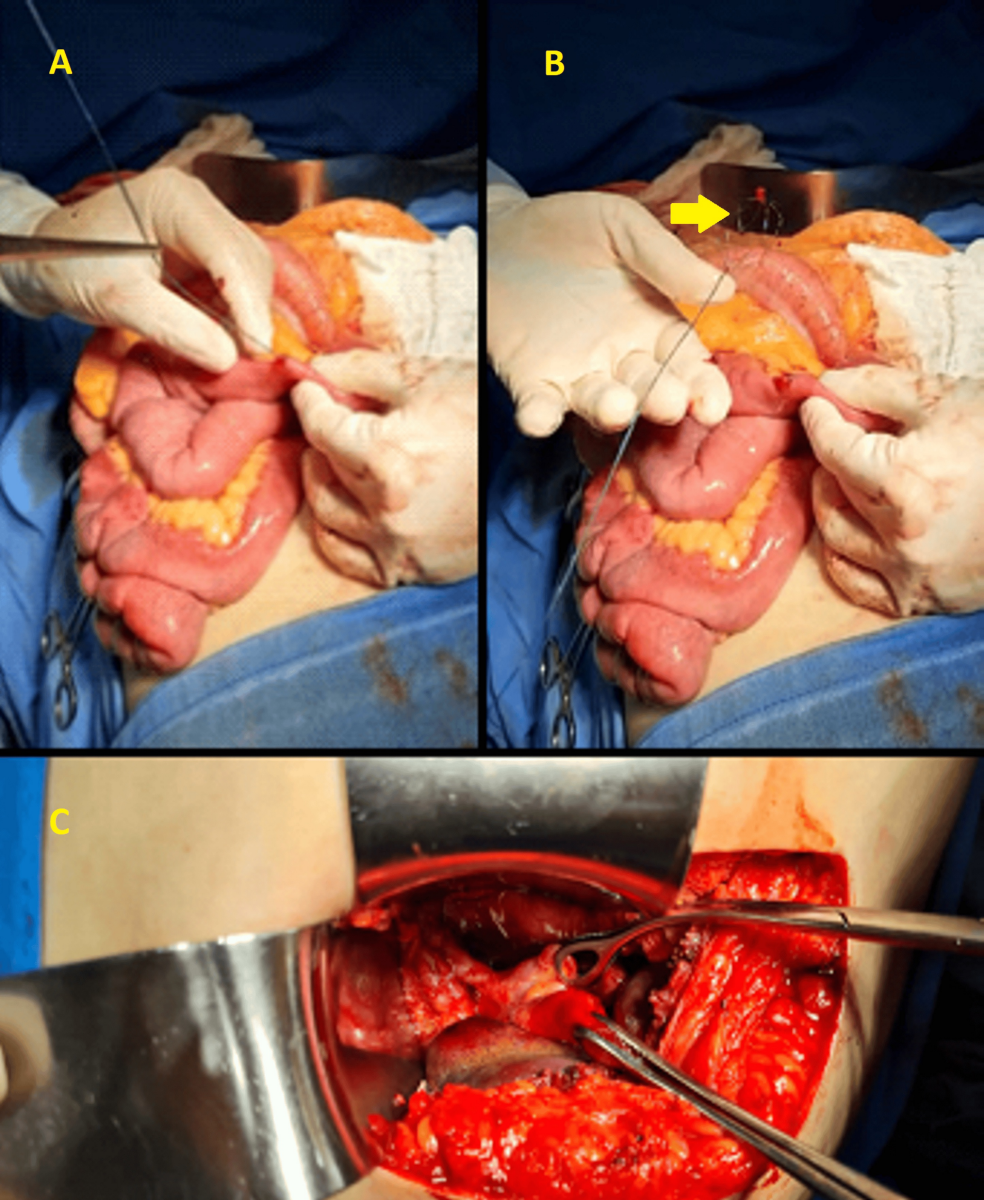
Laparotomy, cholecystectomy, and removal of stent, stone, and basket via duodenotomy and CBD opening (A) Palpation and removal of the basket wire through the duodenum. (B) Dormia basket removed without calculi. (C) Removal of the stone through the opening in the CBD. CBD: common bile duct

The endoscopist then recommended a later endoscopic approach, removing the basket, stone, and prosthesis. However, the surgeon of the case opted for open surgery two days later and cholecystectomy, removal of the prosthesis, stone, and basket by duodenotomy, and CBD opening (Figure 11).

The patient’s evolution was satisfactory, and she was discharged without other complications.

## Discussion

ERCP is a diagnostic and therapeutic procedure commonly used for biliary tract clearance, mainly for removing CBD stones and biliary prosthesis placement in cases of malignant or benign obstruction [7]. Despite a high overall success rate (approximately 95-98%), ERCP-related complications occur in 6-8% of cases. In a study involving 16,855 patients, 1,154 experienced complications (6.85%), and 55 died (0.33%). Mild-to-moderate complications occurred in 872 (5.17%) patients, whereas severe events occurred in 282 (1.67%), pancreatitis in 585 (3.47%), 242 (1.44%), bleeding in 226 (1.34%), and perforations in 101 (0.60%). Cardiovascular and anesthetic complications occurred in 173 (1.33%), with nine fatalities (0.07%) [6,8,9]. The literature offers abundant data on ERCP complications [9]; however, the risks remain significantly lower compared to surgical management of CBD stones. Over recent decades, the treatment of choledocholithiasis has evolved significantly with advances in endoscopic techniques.

This case involved choledocholithiasis with an impacted stone that was large and disproportionate to the caliber of the distal CBD. Despite a wide papillary sphincterotomy and dilation of the distal CBD using a 15-mm balloon catheter, stone extraction was unsuccessful. Urgent mechanical lithotripsy was attempted; however, rupture of the proximal wire of the Dormia basket occurred, making removal of the device impossible. A second attempt was made after repeat dilation of the distal CBD with a 15-mm balloon catheter, but it was also unsuccessful. A plastic biliary stent was then placed. The point of rupture was located at the junction between the nitinol and steel components of the basket rod. This interface may represent a potential point of structural weakness in nitinol baskets and should be taken into consideration when such materials are used.

In cases of disproportion between the size of the large calculi and the narrowing distal portion of the CBD, the endoscopist may opt for some strategies to avoid the imprisonment of the basket and mechanical lithotripsy [10-12]. One is to leave the plastic biliary stent for three to four months and make a new attempt to withdraw. Another option is to perform early cholangioscopy-guided electrohydraulic lithotripsy or laser fragmentation of the stones [13].

There were three possible approaches to resolve this case. The ideal strategy would have been delayed cholangioscopy with laser lithotripsy, followed by removal of the basket, stone, and stent. Alternatively, the case could have been managed laparoscopically through cholecystectomy, choledocholithotomy, and extraction of the basket, stone, and stent. However, definitive treatment was achieved via laparotomy, with cholecystectomy, choledocholithotomy, and removal of the basket, stone, and stent.

## Conclusions

We report a case of choledocholithiasis in which mechanical lithotripsy resulted in rupture of the proximal nitinol rod of a Dormia basket, making stone removal via ERCP difficult and requiring surgical intervention. The most appropriate approach would have been delayed cholangioscopy with laser lithotripsy, followed by the basket, calculi, and stent removal. Alternatively, laparoscopic management with cholecystectomy, choledocholithotomy, and removal of the basket, calculi, and stent could have been considered. However, the case was ultimately resolved through open laparotomy, including cholecystectomy, choledocholithotomy, and removal of all retained elements.
